# NEURL4 regulates the transcriptional activity of tumor suppressor protein p53 by modulating its oligomerization

**DOI:** 10.18632/oncotarget.18699

**Published:** 2017-06-27

**Authors:** Monica Cubillos-Rojas, Taiane Schneider, Ramon Bartrons, Francesc Ventura, Jose Luis Rosa

**Affiliations:** ^1^ Departament de Ciències Fisiològiques, Campus de Bellvitge, Institut d’Investigació Biomèdica de Bellvitge (IDIBELL), Universitat de Barcelona, L’Hospitalet de Llobregat, Barcelona E-08907, Spain

**Keywords:** NEURL4, p53, oligomerization, tetramerization, HERC2

## Abstract

p53 is a transcription factor that regulates important cellular processes related to tumor suppression, including induction of senescence, apoptosis, and DNA repair as well as the inhibition of angiogenesis and cell migration. Therefore, it is critical to understand the molecular mechanism that regulates it. p53 tetramerization is a key step in its activation process and the regulation of this oligomerization, an important control point. The E3 ubiquitin ligase HERC2 controls the p53 transcriptional activity by regulation of its oligomerization state. HERC2-interacting proteins such as the adaptor-like protein with six neuralized domains NEURL4 are also candidates to regulate p53 activity. Here, we demonstrate the existence of an interaction network between NEURL4, HERC2 and p53 proteins. We report a functional interaction between NEURL4 and p53, involving the C-terminal region of p53 and the neuralized domains 3 and 4 of NEURL4. Through this interaction, NEURL4 regulates the transcriptional activity of p53. Thus, NEURL4 depletion reduced the transcriptional activity whereas NEURL4 overexpression increased it. In both cases, p53 stability was not affected. Although NEURL4 may interact with p53 independently of the E3 ubiquitin ligase HERC2, we observed that both proteins are needed to regulate the transcriptional activity of p53. Clonogenic assays confirmed the functional relevance of this interaction observing a decrease in cell growth by NEURL4 overexpression correlated to the increase of cellular cycle inhibitor p21 by p53 activation. Under these conditions, NEURL4 activated p53 oligomerization. All these findings identify NEURL4 as a novel regulator of the p53’s signaling.

## INTRODUCTION

The tumor suppressor p53 plays a central role in coordinating cellular responses to stress. p53 functions as a transcription factor and, although some effects may be independent of its transcriptional activity, the p53-controlled transactivation of target genes is an essential feature in the stress response pathway, determining whether cells respond by cycle arrest, apoptosis, senescence, autophagy, metabolism and/or DNA repair. p53’s ubiquitylation, mediated by E3 ubiquitin ligases and its further proteasomal degradation, maintains low levels of p53 in unstressed cells. Stress signals, such as DNA damage, oncogene activation and hypoxia, promote p53 activation. This activation of p53 generally consists in its stabilization, binding to a sequence-specific DNA, and recruitment of the transcriptional machinery to activate the transcription of p53 target genes. During these sequential steps, p53 activation is modulated by different post-translational modifications, including ubiquitylation, phosphorylation, acetylation, sumoylation, methylation, and neddylation. p53 controls the expression of genes and miRNAs affecting many important cellular processes including proliferation, apoptosis, autophagy, DNA repair, metabolism, and cell migration. Many of these processes are essential to a variety of human pathologies and conditions extending beyond cancer, including ischemia, neurodegenerative diseases, stem cell renewal, aging, and fertility [1–7].

The p53 signaling is inactivated in most cancers. Thus, the *TP53* gene is deleted or mutated in approximately 55% of sporadic human cancers while p53 signaling is disrupted by alterations to its many regulators and/or targets in the remaining tumors [5, 8, 9]. Therefore, it is essential to understand the molecular mechanism by which p53 is regulated. Post-translational modifications of p53 are important in modulating its tumor suppressive functions [10]. During the last years, oligomerization/tetramerization of p53 has also emerged as a critical event in order to regulate the transcription of target genes, necessary to inhibit the tumor growth [11, 12]. Impaired oligomerization of p53 is associated with tumor’s progression such as it happens in some cases of patients with Li-Fraumeni syndrome and Li-Fraumeni-like syndromes [12–14].

The E3 ubiquitin ligase HERC2 interacts with p53 and regulates its transcriptional activity by stimulating p53’s oligomerization [15]. Knowing the regulation of this step could be useful as an alternative way to restore p53 activity in cells with non-mutated p53. Proteins interacting with HERC2 are candidates to regulate p53 oligomerization. The adaptor-like protein with six neuralized domains NEURL4 is a HERC2-interacting protein [16, 17]. In association with HERC2, NEURL4 has been identified as a modulator of centrosome architecture, through the interaction with the centrosomal protein CP110 [17, 18], and of Notch signaling through the endosomal pathway [19]. Although HERC2 has been involved in other cellular processes such as DNA repair or cell cycle progression [20], there is very few information about other cellular functions in which NEURL4 could participate. We asked whether NEURL4 can interact with p53 and participate in processes regulated by p53. In this study, we report a new function for NEURL4. We demonstrate that NEURL4 forms a complex with p53 regulating its transcriptional activity, and affecting the expression of genes such as *p21, p53R2*, or *p53AIPI.* NEURL4’s overexpression is enough to inhibit cellular growth. Furthermore, cross-linking experiments indicated that NEURL4 regulates the oligomerization of p53. These results demonstrate that NEURL4 is a key component in p53 regulation.

## RESULTS

### Interaction network between NEURL4, HERC2 and p53 proteins

**Figure 1:**
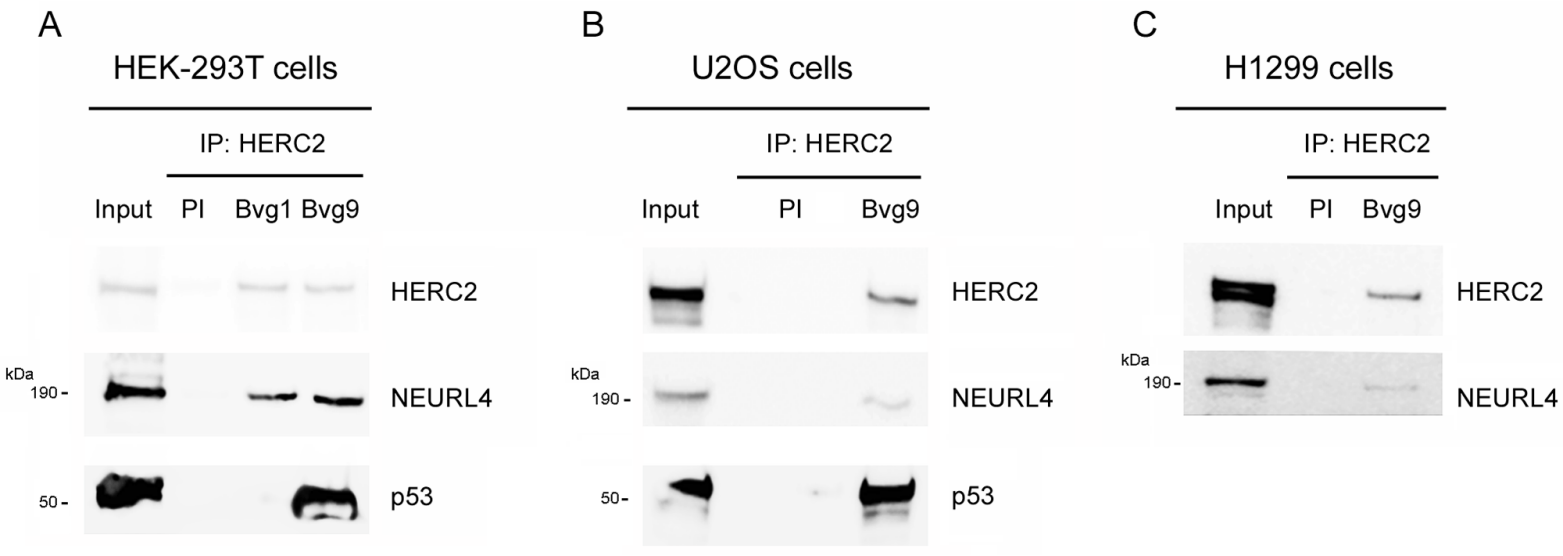
Interaction network between NEURL4, HERC2, and p53 proteins **(A)** Supernatants (input) of lysates from HEK-293T cells were immunoprecipitated (IP) using two different antibodies against HERC2 (Bvg1 and Bvg9) and analyzed by immunoblotting with antibodies against the indicated proteins. Preimmune serum (PI) was used as negative control. Similar experiments were performed in U2OS cells **(B)** and H1299 cells **(C)**.

**Figure 2:**
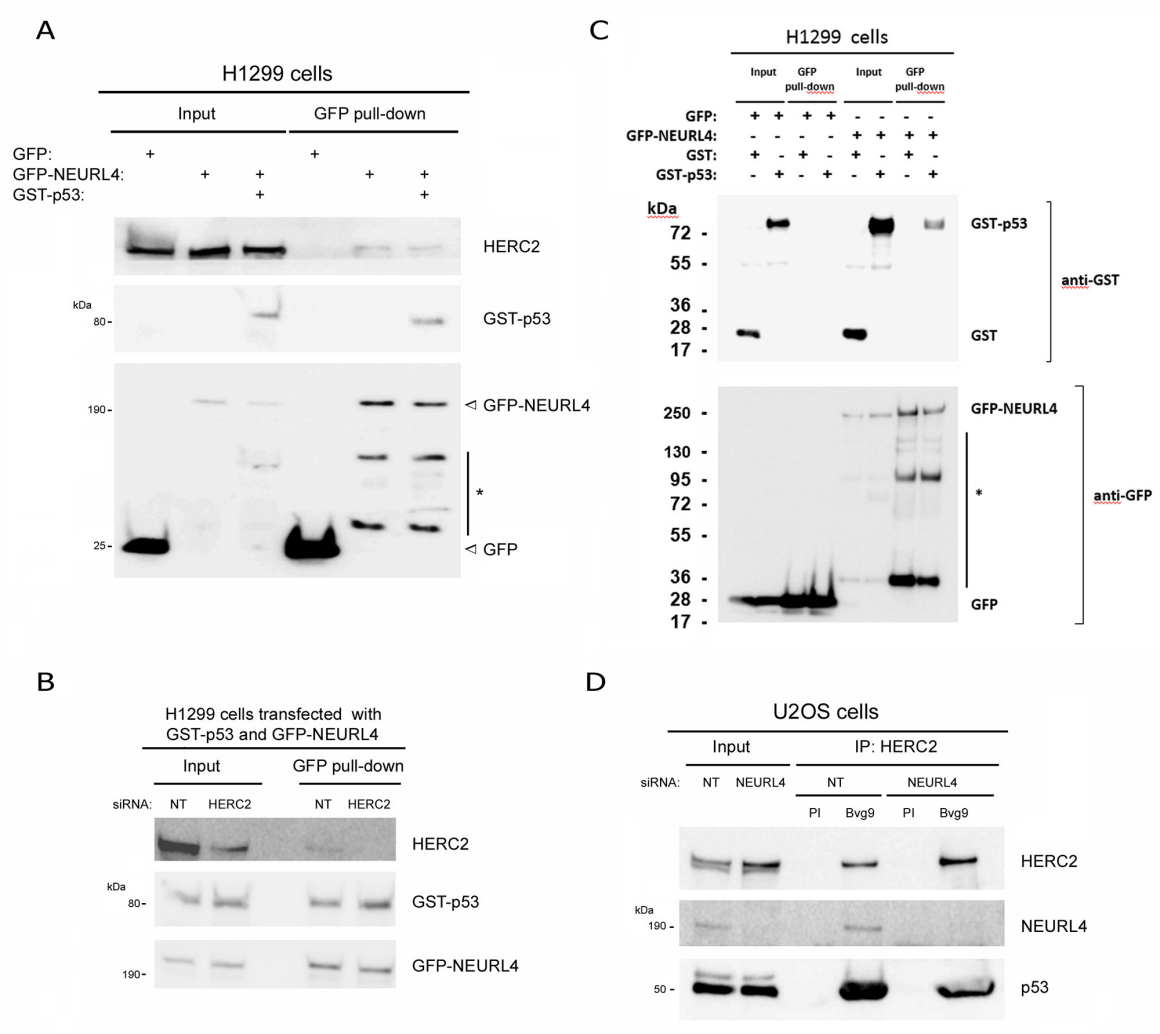
Characterization of the interaction between NEURL4, HERC2, and p53 proteins **(A and C)** The specificity of the interaction between NEURL4, HERC2 and p53 was tested in p53-null H1299 cells with pull-down experiments. Cells were transfected with GFP, GFP-NEURL4, GST and GST-p53 plasmids. 48 h later, cells were lysed and centrifuged. Supernatants (Input) were incubated with GFP-Trap, proteins retained on the resin were analyzed by immunoblotting with antibodies against the indicated proteins. Asterisk (*) means degradation products. **(B)** Interaction between NEURL4 and p53. H1299 cells were transfected with non-targeting (NT) or HERC2 siRNAs and GFP-NEURL4 and GST-p53 plasmids. 72 h later supernatants were incubated with GFP-Trap and analyzed as above is indicated. **(D)** Interaction between HERC2 and p53 independently of NEURL4. U2OS cells were transfected with non-targeting (NT) or NEURL4 siRNAs. 72 h later supernatants were immunoprecipitated with anti-HERC2 (Bvg9) polyclonal antibody and analyzed by immunoblotting as above is indicated. Preimmune serum (PI) was used as IgG antibody control.

NEURL4 interacts with the E3 ubiquitin ligase HERC2 [16, 17] and HERC2 is one of binding partners of p53 [15]. We wondered about the existence of an interaction network between these three proteins. To analyze this point, we performed immunoprecipitation experiments in human embryonic kidney 293T (HEK-293T) cells with two different antibodies against HERC2. Using an antibody against the amino terminus of HERC2 (Bvg9), we observed the coimmunoprecipitation of NEURL4 and p53 (Figure 1A) which indicated an interaction between HERC2 and NEURL4 and p53 proteins. We confirmed these results in human osteosarcoma U2OS cells (Figure 1B), showing that this interaction is not restricted to HEK-293T cells. When we used an antibody against the carboxyl terminus of HERC2 (Bvg1), the coimmunoprecipitation of p53 was lost whereas the interaction between HERC2 and NEURL4 maintained (Figure 1A) suggesting an interaction between HERC2 and NEURL4 independent of p53. This observation was later confirmed in human non-small cell lung carcinoma H1299 cells which do not express p53. In these cells, the interaction between HERC2 and NEURL4 was also observed (Figure 1C). To understand this interaction network, we analyzed the immunoprecipitates of NEURL4 but the commercial antibody did not immunoprecipitate NEURL4. Alternatively, we expressed GFP-NEURL4 fusion protein in H1299 cells and we performed pull-down experiments using GFP-Trap, a GFP-binding protein coupled to agarose beads. We observed as GFP-NEURL4 interacted with endogenous HERC2 in the absence of p53 (Figure 2A, lane 5) demonstrating the existence of a NEURL4-HERC2 complex. Interestingly, in cells transfected with GFP-NEURL4 and GST-p53, we observed how GST-p53 also pull-downs with NEURL4 and HERC2 (Figure 2A, lane 6). We repeated these experiments after HERC2’s knockdown and we observed how GST-p53 interacts with NEURL4 in the absence of HERC2 (Figure 2B). Pull-down experiments with GFP-alone or GFP-NEURL4 confirmed the specificity of this interaction (Figure 2C). Altogether, these experiments indicate the formation of a NEURL4-p53 complex. To determine if NEURL4 is necessary for the HERC2-p53 interaction, we transfected siRNA to deplete NEURL4 in U2OS cells and, 72 hours post-transfection, we analyzed this interaction by immunoprecipitation using an antibody against HERC2. Although in less extension, HERC2-p53 interaction was also observed after NEURL4 depletion (Figure 2D). These data together with the interaction between HERC2 and NEURL4 independently of p53 (Figure 1B and 2A) and the interaction between NEURL4 and p53 independently of HERC2 (Figure 2B), demonstrate the existence of an interaction network between NEURL4, HERC2 and p53 proteins.

**Figure 3:**
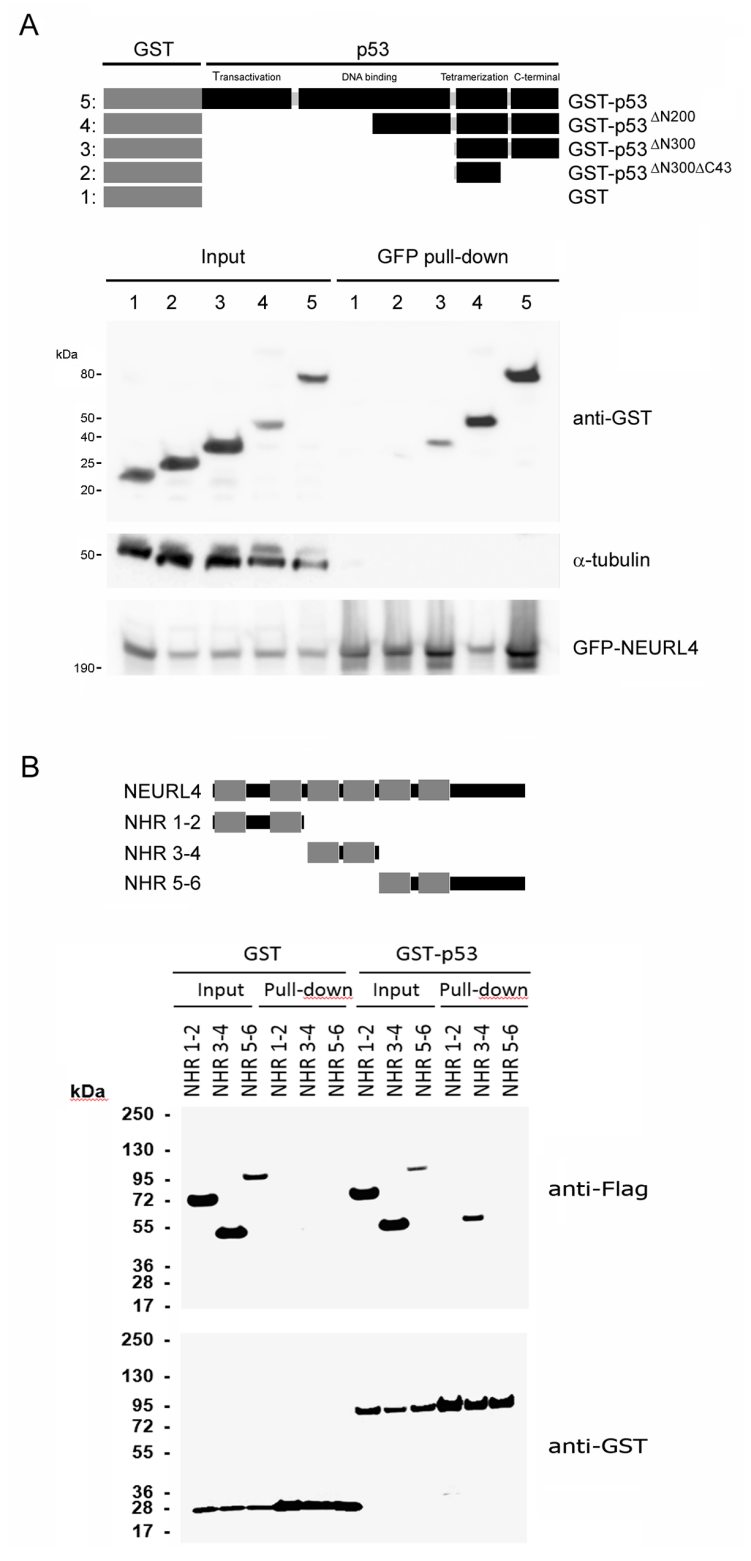
Interaction domains between p53 and NEURL4 **(A)** H1299 cells were transfected with GFP-NEURL4 and the indicated GST-p53 constructs expressing different regions of p53 fused to GST. Domains of p53 are indicated. GST construct was used as negative control. 48 h later, cells were lysed and centrifuged. Supernatants (Input) were incubated with GFP-Trap. Proteins retained on the resin were analyzed by immunoblotting with antibodies against the indicated proteins. α-tubulin was analyzed as control of unspecific binding. **(B)** H1299 cells were transfected with GST or GST-p53 and the indicated constructs expressing different regions of NEURL4. Neuralized domains (NHR) of NEURL4 are indicated. 48 h later, cells were lysed and centrifuged. Supernatants were incubated with Glutathione-Sepharose. Proteins retained on the resin were analyzed by immunoblotting with antibodies against the indicated proteins.

To map NEURL4-p53 interaction, we expressed GFP-NEURL4 and different GST-p53 fusion proteins (Figure 3A) in H1299 cells, and we performed pull-down experiments using GFP-Trap. We observed that GFP-NEURL4 binds to all the GST-p53 fusion proteins expressed except for the GST-p53^∆N300∆C43^ fusion protein (Figure 3A), indicating that the last 43 amino acid residues of p53 are essential for the interaction with NEURL4. Levels of α-tubulin protein were analyzed as negative control. Later, we expressed GST or GST-p53 with different constructs ofNEURL4 (Figure 3B). Pull-down experiments using glutathione-Sepharose showed that p53 binds with more affinity to the neuralized domains (NHR) 3 and 4 of NEURL4 (Figure 3B).

### NEURL4 stability and ubiquitin ligase activity of HERC2

**Figure 4:**
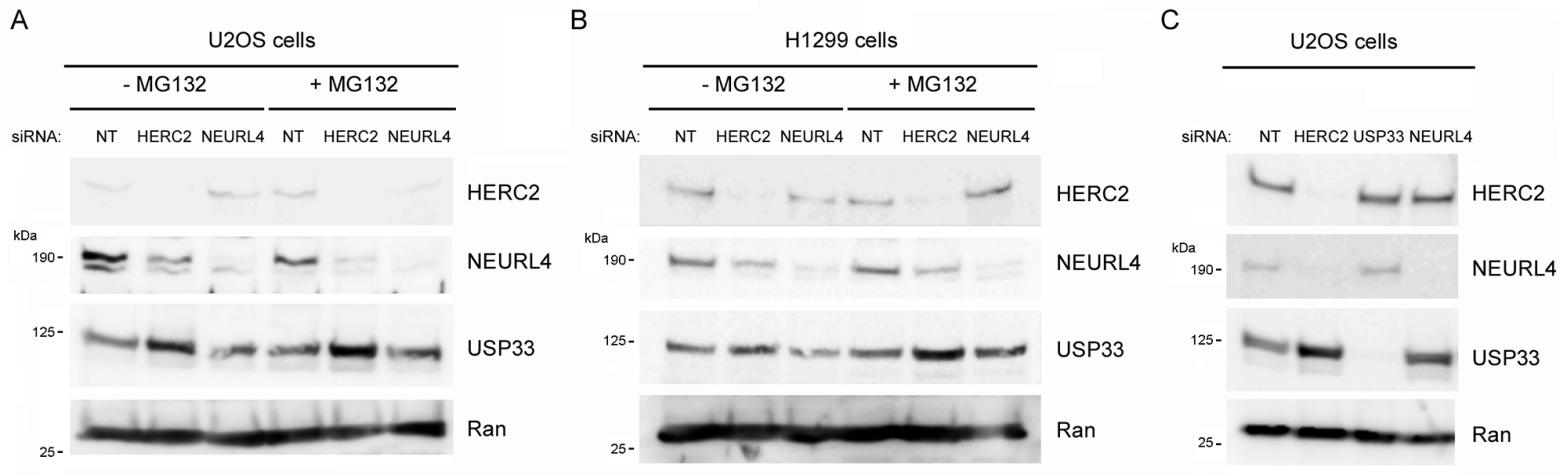
Ubiquitin ligase activity of HERC2 and NEURL4 stability U2OS cells **(A and C)** or H1299 cells (B) were transfected with non-targeting (NT), HERC2, NEURL4 or USP33 siRNAs. 72 h later, lysates were analyzed by immunoblotting with antibodies against the indicated proteins. Before lysis, cells were treated with the proteasome inhibitor MG132 for 6 h. Ran protein was analyzed as loading control.

The interaction between NEURL4 and HERC2 suggested that NEURL4 could regulate the E3 ubiquitin ligase activity of HERC2. To answer this question, we analyzed the levels of USP33, a specific substrate of the ubiquitin ligase activity of HERC2 [21]. We depleted U2OS cells of HERC2 or NEURL4 using RNA interference. 72 hours post-transfection, cells were treated or not with the proteasome inhibitor MG132 and the levels of USP33 were analyzed. Knockdown of HERC2 increased the levels of USP33 (Figure 4A, lane 2), confirming that HERC2 functions as an ubiquitin ligase for USP33. Similar results were obtained when the cells were treated with MG132 (Figure 4A, lane 5). We also observed that HERC2 knockdown affects NEURL4 stability. A decrease of NEURL4 amount was observed (Figure 4A, lanes 2 and 5). When we analyzed the effect of NEURL4 depletion, USP33 amount was unaffected (Figure 4A, lanes 3 and 6 respect to lanes 1 and 4, respectively), indicating that under these conditions NEURL4 did not regulate the ubiquitin ligase activity of HERC2. Similar results were observed in H1299 cells (Figure 4B). Additionally, we tested if USP33 could be involved in the regulation of NEURL4 stability. Endogenous levels of NEURL4 protein were not affected by USP33 depletion (Figure 4C). Altogether, these data show that HERC2 stabilizes NEURL4 protein and that NEURL4 does not affect the ubiquitin ligase activity of HERC2 on USP33.

### NEURL4 regulates the transcriptional activity of p53

**Figure 5:**
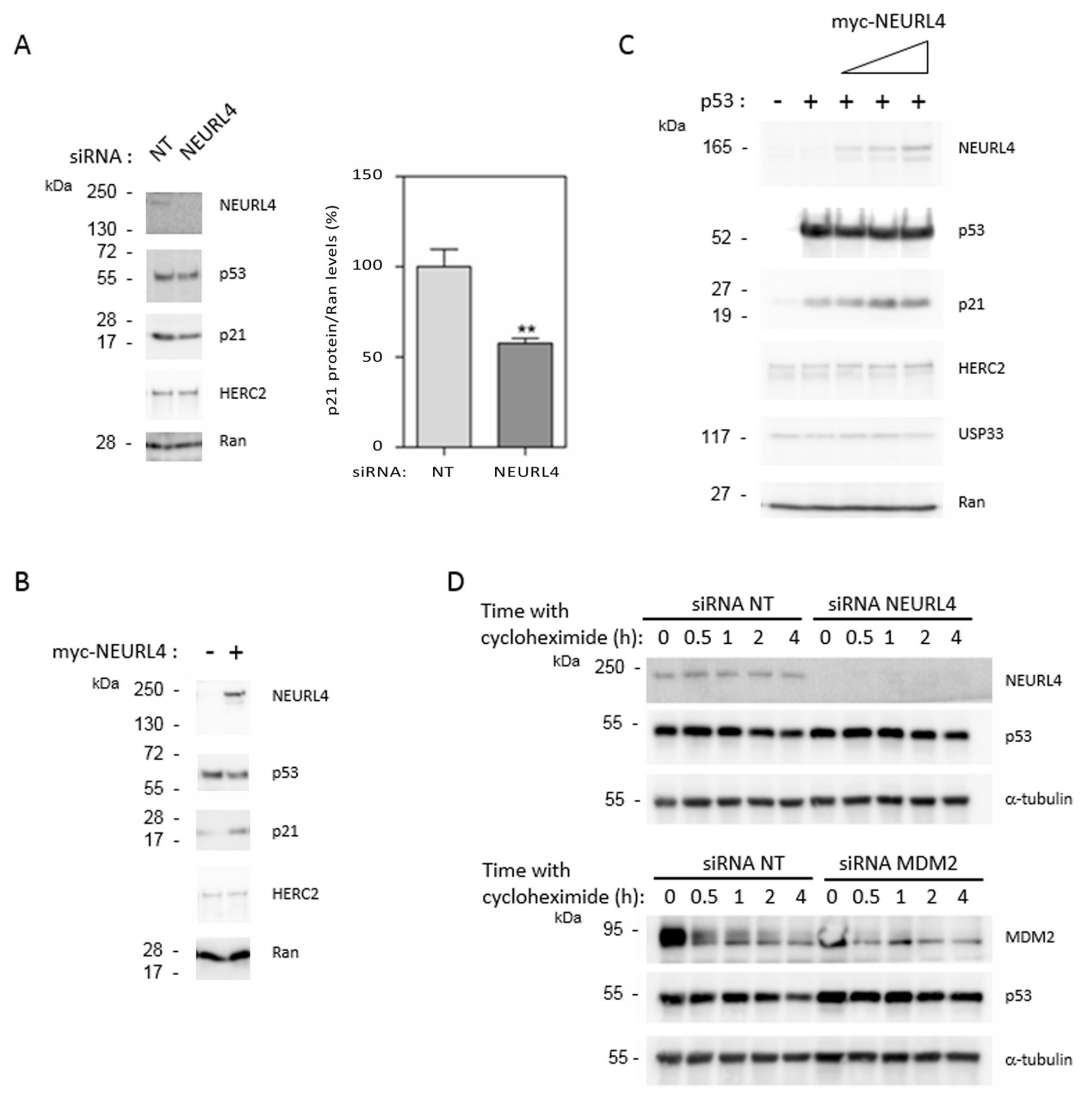
NEURL4 regulates the levels of p21 protein **(A and D)** U2OS cells were transfected with non-targeting (NT), NEURL4 or MDM2 siRNAs. 72 h later, lysates were analyzed by immunoblotting with antibodies against the indicated proteins. Levels of p21 protein were quantified and normalized with respect to Ran levels. When it is indicated, cells were treated with cycloheximide (20 μg/ml) for the indicated times. **(B)** U2OS cells were transfected with pcDNA3 (-) or myc-NEURL4 (+) plasmids. **(C)** H1299 cells were transfected with 0.5 μg of p53 construct and increasing amounts (0.5, 1 and 2 μg) of myc-NEURL4 constructs. 48 h later, lysates from transfected cells were analyzed by immunoblotting with antibodies against the indicated proteins. Ran protein was analyzed as loading control. Data are expressed as mean ± S.E.M. Statistical analysis was carried out as described under “Materials and Methods”. The differences are shown with respect to NT siRNA. **, p< 0.01.

To identify whether NEURL4 could regulate p53 activity, we analyzed the protein levels of p21. Whereas p53 levels were not significantly modified, a great decrease in p21 levels was observed after NEURL4 depletion in U2OS cells (Figure 5A), suggesting that NEURL4 could be involved in the regulation of p53 transcriptional activity. To confirm this observation, we analyzed if NEURL4 overexpression is enough to increase the levels of p21 protein. U2OS cells were transfected with a plasmid expressing myc-NEURL4. After 48 hours of transfection, the levels of p21 protein were analyzed. We observed an increase of the levels of p21 protein when NEURL4 was overexpressed (Figure 5B). These results were also confirmed in other cellular type. H1299 cells were transfected with a p53’s plasmid and with increasing amounts of a plasmid expressing myc-NEURL4. After 48 hours of transfection, the levels of p21 protein were analyzed. We observed a correlation between the increase of the myc-NEURL4 amount and the increase of p21 levels (Figure 5C). In agreement with the previous observations (Figure 4), levels of HERC2 or USP33 were not affected (Figure 5A–5C). To conclude that these effects are independent on the p53 stability, experiments were performed in U2OS cells using the translational inhibitor cycloheximide. Time course experiments with cycloheximide after siRNA transfection showed that the stability of p53 was not significantly modified by NEURL4 depletion whereas it was increased by depletion of MDM2, a well-known ubiquitin ligase for p53 (Figure 5D).

**Figure 6:**
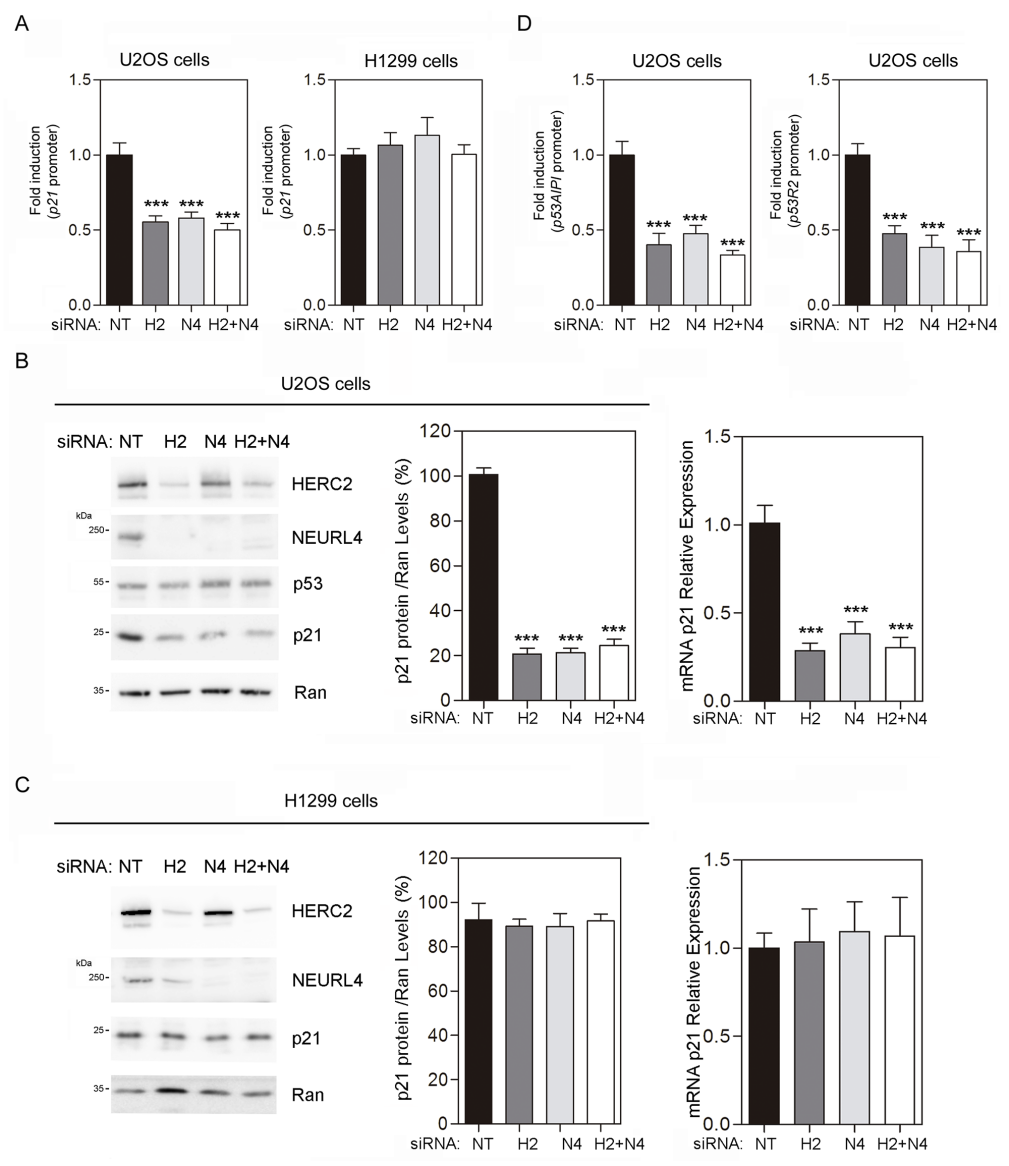
NEURL4 regulates the transcriptional activity of p53 **(A and D)** U2OS cells or H1299 cells were transfected with non-targeting (NT), NEURL4 (N4) or HERC2 (H2) siRNAs and the luciferase reporter indicated (*p21*, *p53AIPI* or *p53R2* promoter). 72 h later, the luciferase activity was quantified as indicated under “Materials and Methods”. **(B)** U2OS cells or **(C)** H1299 cells were transfected with non-targeting (NT), NEURL4 (N4) or HERC2 (H2) siRNAs. RT quantitative PCR (RT-qPCR) analysis was performed to analyse gene expression of *p21*. The levels of *p21* expression were quantified and normalized with respect to *GAPDH* gene expression (right panels in B and C). Lysates from these cells were also analyzed by immunoblotting with antibodies against the indicated proteins (left panels in B and C). Levels of p21 protein were quantified and normalized with respect to Ran levels (central panels in B and C). Data are expressed as mean ± S.E.M. Statistical analysis was carried out as described under “Materials and Methods”. The differences are shown with respect to NT siRNA. **, p< 0.01; ***, p< 0.001.

These findings would suggest that a NEURL4-dependent mechanism is involved in the transcriptional regulation of *p21* by p53. To analyze this, luciferase reporter assays were performed in U2OS cells transfected with the *p21* promoter. We observed a decrease in *p21* promoter activity after NEURL4 depletion (Figure 6A, left). Endogenous *p21* mRNA levels were also decreased by NEURL4 knockdown in U2OS cells (Figure 6B, right) correlating with the reduction of p21 protein levels (Figure 6B, left and center). Similar decreases were observed using HERC2 siRNA or both siRNAs conjointly (Figure 6A and 6B). In parallel experiments, no variations were observed in p53-null H1299 cells (Figure 6A, right, and 6C). These results were also confirmed with other genes regulated by p53 such as *p53AIP1* and *p53R2*. Thus, NEURL4 depletion reduced significantly the promoter activity of *p53AIP1* and *p53R2* (Figure 6D).

### NEURL4 regulates p53 oligomerization and cell growth

**Figure 7:**
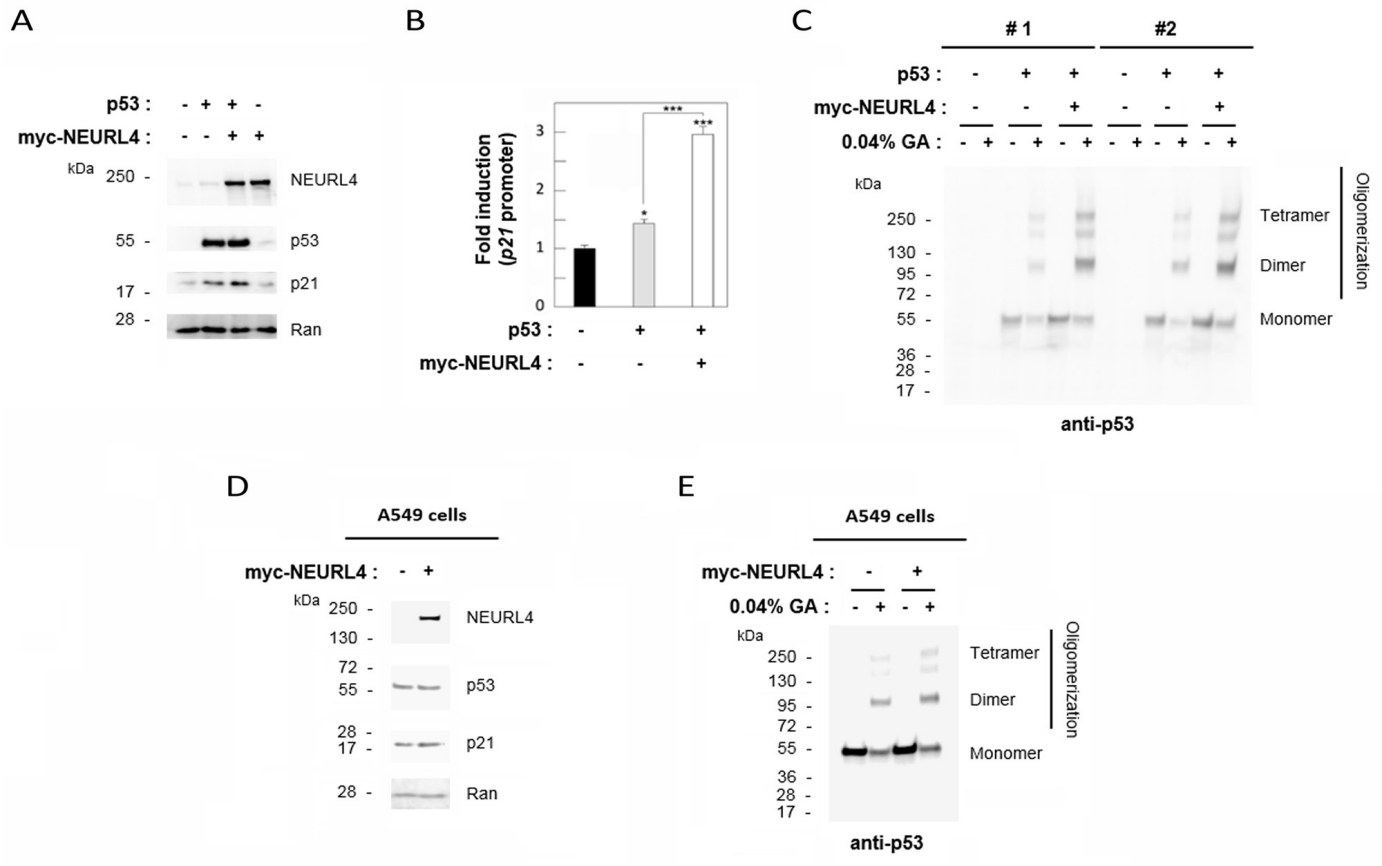
NEURL4 regulates p53 oligomerization H1299 **(A-C)** and A549 **(D and E)** cells were transfected with pcDNA3 (negative control: -) and the indicated plasmids. **(A and D)** 24 h later, lysates were analyzed by immunoblotting with antibodies against the indicated proteins. Ran protein was analyzed as loading control. **(B)** Under the same conditions, luciferase activity of *p21* promoter was analyzed as indicated under “Materials and Methods”. **(C and E)** Lysates from cells transfected with the indicated plasmids were incubated with glutaraldehyde (GA) at 0.04% on ice for 30 min, and p53 oligomerization was analyzed by immunoblotting with anti-p53 antibody as indicated under “Materials and Methods”. Data are expressed as mean ± S.E.M. Statistical analysis was carried out as described under “Materials and Methods”. *, p< 0.05; ***, p< 0.001.

The interaction network between NEURL4, p53 and HERC2 proteins (Figure 1 and 2) and the similar regulation of p53 transcriptional activity by NEURL4 and HERC2 (Figure 6) led us to analyze whether NEURL4 could regulate p53 activity by a mechanism similar to HERC2. Because we had previously demonstrated that HERC2 modulates p53 activity by regulating its oligomerization [15], we decided to analyze if p53 oligomerization was also regulated by NEURL4. To this end, we overexpressed myc-NEURL4 in H1299 cells. Expression of this plasmid significantly increased the levels of p21 protein when p53 was co-expressed (Figure 7A) and correlating with the increase of the p53 transcriptional activity (Figure 7B). Under these conditions, p53 oligomerization was analyzed using a cross-linking assay [15, 22]. We observed a great increase in p53 oligomerization in cells overexpressing NEURL4 (Figure 7C). Similar results were observed in p53 harboring cells like A549 cells (Figure 7D and 7E). Altogether these data demonstrate that NEURL4 expression is sufficient to regulate p53 oligomerization and induce its transcriptional activity.

**Figure 8:**
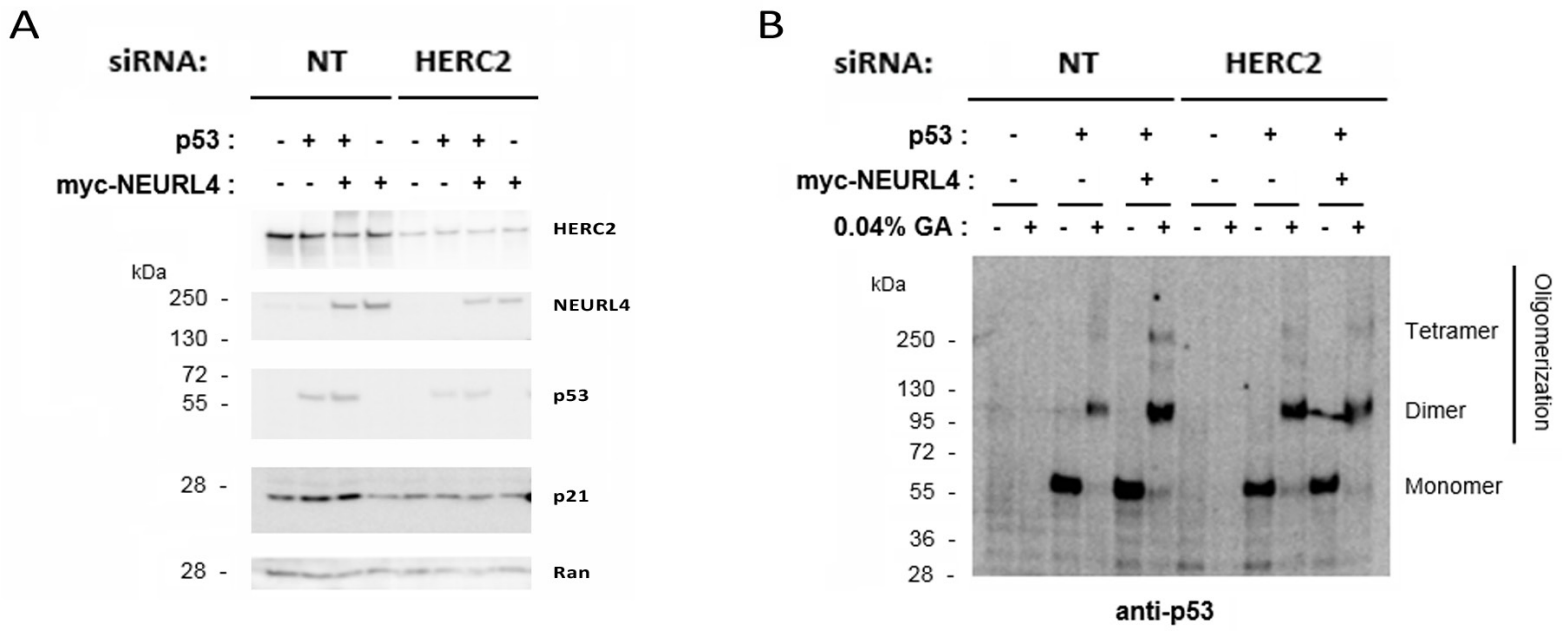
HERC2 is required for the regulation of p53 activity mediated by NEURL4 H1299 cells were transfected with plasmids and siRNAs which are indicated. 72 h later, lysates were analyzed by immunoblotting with antibodies against the indicated proteins **(A)** or by protein cross-linking assay **(B)** as described under “Materials and Methods”.

Conjoint depletion of NEURL4 and HERC2 have an effect similar on the regulation of p53 transcriptional activity that their depletions individual (Figure 6), suggesting that both proteins are necessary for this regulation. Having in mind this, we asked whether HERC2 was required for the increase in p21 levels mediated by overexpression of NEURL4 (Figure 7). To analyze this point, the effect of HERC2 depletion in the p21 protein levels was analyzed in H1299 cells expressing myc-NEURL4 and/or p53. We observed that HERC2 knockdown inhibited the increase of levels of p21 mediated by NEURL4 overexpression when p53 was co-expressed (Figure 8A, see lane 7 respect to lane 3). Interestingly, when we analyzed p53 oligomerization, we also observed that HERC2 knockdown inhibited the p53 oligomerization mediated by NEURL4 overexpression (Figure 8B, see lane 12 respect to lane 6).

**Figure 9:**
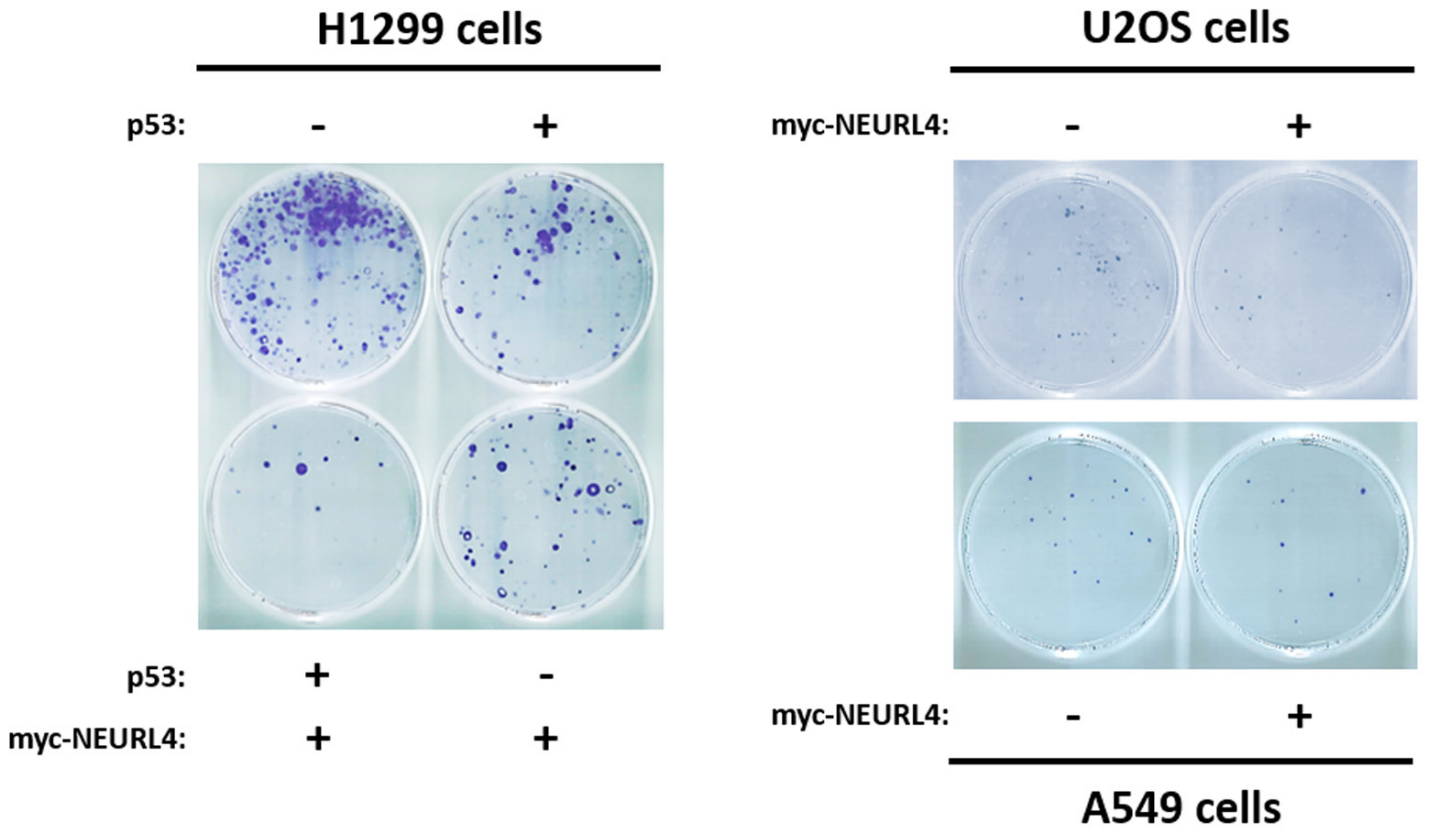
NEURL4 regulates cell growth Colony formation assays were performed in cells transfected with the indicated plasmids. 24 h later, G418 antibiotic was added (at 400, 500 or 700 μg/ml for U2OS, H1299 or A549 cells, respectively) and maintained for 12-15 days. After this time, cells were stained with crystal violet as indicated under “Materials and Methods”.

Finally, to show the functional importance of the regulation of p53 activity by NEURL4, we decided to analyze cellular processes regulated by p53 such as cell growth. To address this, we analyzed the growth of p53-null H1299 cells using a clonogenicity assay. As expected, the expression of p53 plasmid in H1299 cells reduced the number of colonies (Figure 9, left panel). Interestingly, a greater decrease in the number of colonies was observed when myc-NEURL4 was also expressed, in agreement with the increase of cell cycle inhibitor p21 (Figure 5C and 7A). Similar results were observed in cells harboring p53 when myc-NEURL4 was overexpressed (Figure 9, right panels).

## DISCUSSION

The tumor suppressor p53 is a transcription factor that regulates genes expression involved in cellular processes linked to growth suppression. Since activation of p53 transcriptional activity requires its oligomerization, regulating this step is an important control point. In humans, p53 mutations that affect its oligomerization state have pathological consequences as observed in patients with Li-Fraumeni syndrome [13, 14]. The ubiquitin ligase HERC2 is one of the few proteins reported to regulate p53 oligomerization [15]. The adaptor-like protein with six neuralized domains NEURL4 is a HERC2-interacting protein [16, 17, 23] that modulates of centrosome architecture through the interaction with the centrosomal protein CP110 [17, 18]. Now, this study reports the existence of an interactions network between NEURL4, HERC2 and p53 proteins. Structurally, our results indicate that each one of these three proteins may interact with each other two independently. Interestingly, NEURL4 (Figure 3A) and HERC2 [15] interact with the same region of p53. Through these interactions, the transcriptional activity of p53 is regulated. Proteins assemble into complexes to exert their functions within the cell. Experiments of size exclusion chromatography previously demonstrated a similar elution profile for HERC2 and NEURL4, being part of a high-molecular mass complex of about 2 MDa [16]. These data together with the observation that the depletion of HERC2 and/or NEURL4 inhibited in a similar extension the transcriptional activity of p53, suggest a coordinated function of these proteins on p53. The interaction of NEURL4 with the C-terminal amino acid residues of p53 enclosing part of its tetramerization domain would suggest the involvement of NEURL4 in p53 oligomerization. This hypothesis could be demonstrated using cross-linked studies with glutaraldehyde. Thus, we show that NEURL4 regulates p53 oligomerization.

Mutations located within the p53 tretamerization domain disrupt p53 oligomerization [12]. Interestingly, p53 acetylation does not occur on p53 mutants that are incapable of forming oligomers because acetyltransferases cannot interact [12]. p53 acetylation is indispensable for its activation [11]. These observations suggest a model for p53 activation in which p53 oligomerization precedes its acetylation and activation [12]. Our findings incorporate HERC2 and NEURL4 to this model functioning like necessary cofactors for p53 oligomerization.

HERC2 through its ubiquitin ligase activity may label proteins such as USP33 with ubiquitin for ubiquitin-dependent proteasomal degradation [21]. Although it has been reported that NEURL4 is ubiquitylated in a HERC2-depedent manner [17], we observe that neither NEURL4 levels neither p53 levels were increased after HERC2 knockdown. In fact, NEURL4 was destabilized in absence of HERC2. These data are in agreement with previous observations [15–17] confirming that HERC2 does not regulate p53 levels and suggesting that HERC2-dependent NEURL4 ubiquitylation must serve a different purpose than regulating NEURL4 levels. NEURL4 could also act as a regulator of the ubiquitin ligase activity of HERC2. In fact, a role as an adaptor bringing substrates to the E6AP and HERC2 ubiquitin ligases had been previously suggested [24]. Although this possibility is attractive, the stability of USP33 levels after NEURL4 depletion would indicate that NEURL4 is probably not involved in processes regulated by the ubiquitin ligase activity of HERC2 such as the modulation of iron metabolism [25, 26] or the response to DNA damage [27–31].

NEURL4 has been recently identified as a member of the superfamily of ADP-ribosyltransferases [23]. However, this activity and their biological implications have not been studied. NEURL4 has been implicated in the centrosome assembly [17, 18] and more recently in Notch signaling [19]. Our results identify a new function for NEURL4 as a regulator of p53 activity. p53 controls the expression of genes and miRNAs affecting many important cellular processes including proliferation, DNA repair, apoptosis, autophagy, metabolism or cell migration [3–5]. NEURL4 through the regulation of p53 activity could be involved in some of these cellular processes. Although more studies will be necessary to know the NEURL4 relevance in these cellular processes, the demonstration that NEURL4 expression regulated cell growth, shows an important and unknown role of NEURL4 in proliferation. These characteristic points out that NEURL4 could function as a tumor suppressor protein. Consequently, deletions of *NEURL4* gene could enhance tumorigenesis in some types of cancer. Interestingly, analysis of cancer genomics data sets from the cBioPortal (http://www.cbioportal.org/) shows a high frequency in *NEURL4* deletions in three different studies (TCGA2015, MICH and TCGA) of prostate cancer [32, 33].

In conclusion, we identify a new function for NEURL4 as a regulator of p53 signaling. We demonstrated that NEURL4 interacts with p53 and regulates its transcriptional activity through modulation of its oligomerization. Our data contribute to clarify the activation model of p53 as well as pointing out pathological consequences of the NEURL4 dysregulation.

## MATERIALS AND METHODS

### Reagents

The following reagents were used: anti-HERC2 monoclonal (BD Biosciences #612366); anti-HERC2 polyclonal (Bvg1 antibodies against residues 4785-4834, and Bvg9 antibodies against residues 1-199) [15]; anti-p21 (C-19), anti-p53 (FL-393), anti-NEURL4 (E-20) (Santa Cruz Biotechnology, Inc.); anti-p53 Ab-5 (DO-7) (Neo Markers); anti-GST monoclonal (GenScript); anti-Ran [34]; anti-α-tubulin (Ab-1) (Calbiochem); anti-USP33 (Proteintech); anti-Flag M2 (Sigma); anti-GFP and anti-c-myc (clone 9E10) (Roche); anti-MDM2 (2A10) (Abcam); Z-Leu-Leu-Leu-al (MG132) (Sigma-Aldrich); horseradish peroxidase-conjugated secondary antibodies; lipofectamine LTX (Invitrogen); cycloheximide (Applichem); protein A-Sepharose and glutathione-Sepharose (GE Healthcare); GFP-Trap_A (ChromoTek); Immobilon-P PVDF transfer membrane (Millipore Corporation); luciferase assay system (Promega); luminescent β-galactosidase detection Kit II (Clontech Laboratories).

### Plasmids and siRNAs

pEGB–p53 constructs (WT, ΔN200, ΔN300 and ΔN300ΔC43), p53 construct (WT), luciferase reporters (p21WAF1, p53AIPI and p53R2), GFP-NEURL4 construct and myc-NEURL4 construct and NEURL4 constructs (full-length, NHR 1-2, NHR 3-4 and NHR 5-6) were kindly provided by Dr Y. Xiong [35], Dr. Y. Zhang [12], Dr Y. Taya [36], Dr. L. Pelletier [17] and Drs. Y. Imai and R. Takahashi [19], respectively. siRNAs targeting the human sequence of HERC2 (H2.2: GACUGUAGCCAGAUUGAAA); NEURL4 (CCAUCAUGCAAGACGGUAA); USP33 (GAUCAUGUGGCGAAGCAUA); MDM2 (UGGU-UGCAUUGUCCAUGGC) and a non-targeting siRNA (NT: UAGCGACUAAACACAUCAA) were purchased from GenePharma.

### Cell culture, transfections and clonogenic assays

HEK-293T, U2OS, A549 and H1299 human cells from ATCC were cultured at 37°C in Dulbecco’s modified Eagle’s medium (DMEM) (Gibco) with 10% fetal bovine serum. The transfection of cells (plasmids and siRNAs) was carried out using calcium phosphate [15] or lipofectamine LTX according to instructions of the fabricant. The final concentration of siRNAs was 100 nM and the total plasmid DNA 2 μg (for lipofectamine LTX) or 4 μg (for calcium phosphate) into a well of a 6-well plate. When it was necessary for the experiments, 10 ng of p53 construct was transfected in H1299 cells. Cells transfected with plasmids were analyzed after 24-48 hours of the transfection. Cells transfected with plasmids and siRNAs or only siRNAs were recovered at 72 h post-transfection. MG132 was added to the cells for 6 h to a final concentration of 10 μM. For clonogenic assays, cells into 6-well plates at 50% confluence were transfected with pcDNA3 and the indicated plasmids using lipofectamine LTX. 24 hours later, the cells were trypsined and seeded (1/10) into 10 cm-diameter plates. 24 hours later, G418 antibiotic was added (at 400, 500 or 700 μg/ml for U2OS, H1299 or A549 cells, respectively) and maintained for 12-15 days. After this time, the cells were stained with crystal violet as previously described [15].

### Cell lysate and immunoblotting

Cells were lysed with NP40 buffer (50 mM Tris-HCl, pH 7.5, 150 mM NaCl, 50 mM NaF, 0.5% NP40) containing protease and phosphatase inhibitors (50 mM β-glycerophosphate, 1 mM sodium vanadate, 1 mM phenylmethylsulfonyl fiuoride, 5 μg/ml leupeptin, 5 μg/ml aprotinin, 1 μg/ml pepstatin A and 100 μg/ml benzamidine). Cell lysates were sonicated on ice and maintained under agitation for 20 min. The lysates were then centrifuged at 13,000 x g at 4°C for 10 min, and the supernatants were collected to be analyzed by electrophoresis and immunoblot using the Tris-acetate PAGE system [37]. Band intensities were analyzed using a gel documentation system (LAS-3000 Fujifilm). Protein levels were normalized with respect to Ran levels and expressed as a percentage of controls.

### Immunoprecipitations and pull-down experiments

For immunoprecipitation (IP), supernatants (input) were incubated with preimmune serum (PI) or with anti-HERC2 polyclonal antibodies (Bvg1 or Bvg9) for 2 h at 4ºC with gentle agitation. Antibodies were immunoprecipitated with protein A-Sepharose for 1 h at 4ºC. Beads were pelleted by centrifugation at 2,500 x g, washed five times with NP40 buffer, and analyzed by electrophoresis and immunoblot as it was indicated above. For pull-down experiments, supernatants (input) were incubated with GFP-Trap or Glutathione-Sepharose beads overnight at 4°C. Pellets were washed five times with NP40 buffer and analyzed by electrophoresis and immunoblot as it was indicated above.

### Protein cross-linking assay

After lysis, the cells were centrifuged and the supernatant was recovered. Glutaraldehyde was added to the supernatant at the indicated concentrations and incubated on ice for 30 min. Cross-linking reaction was stopped by adding sample buffer (1x final concentration), and the samples were analyzed by electrophoresis and immunoblot, similarly as it was described previously [15].

### Luciferase assay

U2OS or H1299 cells were transfected with the corresponding reporter (0.4 μg of reporter and 0.2 μg CMV-βgal). Luciferase activity was quantified using the Luciferase Assay System (Promega). Luciferase values were normalized using β-galactosidase activity measured using the Luminescent β-galactosidase Detection Kit II (Clontech Laboratories). Luminescent levels are expressed as a percentage of controls.

### Reverse transcriptase quantitative PCR (RT-qPCR) analysis

Total RNA was isolated from transfected cells using Trisure reagent (Bioline) according to the manufacturer’s protocol. 2 μg of total RNA were reverse-transcribed using the high capacity cDNA Reverse Transciption kit (Applied Biosystems). Quantitative PCR was carried out using ABI Prism 7900 HT Fast Real-Time PCR System, and commercially available human TaqMan assays (Applied Biosystems) were used to quantify gene expression of CDKN1A (p21) (Hs00355782_m1) and the housekeeping gene GAPDH (HS99999905_m1) was used to normalize them. PCR data were captured and analyzed using the Sequence Detector software (SDS version 2.3; Applied Biosystems).

### Statistical analysis

Results are expressed as mean ± S.E.M. Data for multiple variable comparisons were analyzed by one-way analysis of variance (ANOVA). For comparison of significance, Dunnet’s test or Tukey’s test were used according to the statistical program GraphPad Prism.
